# Giant supra and retrosellar glioependymal cyst presenting with only precocious puberty. Clinical study and review of the literature

**DOI:** 10.1016/j.ijscr.2024.109360

**Published:** 2024-02-21

**Authors:** Hassan Kadri, Mazen Dughly, Mohamad Shehadeh Agha, Ghiath Hamed, Raed Abouharb, Rostom Mackieh

**Affiliations:** a Department of Neurosurgery, Children's University Hospital, Faculty of Medicine, Damascus University, Syria; b Department of Neurology, Faculty of Medicine, Damascus University, Syria; c Department of Neuroradiology, Damascus Hospital, Syria; d Amana Pathology Lab, Damascus, Syria

**Keywords:** Glioependymal cyst, Precocious puberty, Neuroendoscopy

## Abstract

**Introduction and importance:**

Intracranial glioependymal cysts are an uncommon type of neuroepithelial cyst and are encountered much less frequently than arachnoid cysts. These cysts primarily manifest within the parenchyma of the brain, although exceedingly rare instances have been reported in the lateral ventricles.

**Case presentation:**

We present a highly unusual case of a glioependymal cyst in a 7-year-old girl. The glioependymal cyst was located in the midline in the suprasellar region and extended to the upper clivus region. Its only manifestation was precocious puberty. We performed endoscopic fenestration of the cyst, leading to a return of hormonal levels to normal and a slight reduction in cyst size.

**Clinical discussion:**

A comprehensive search of the Medline database revealed only a few documented cases of glioependymal cysts (fewer than 30 cases). Remarkably, the majority (if not all) were located laterally rather than in the midline of the brain. Endoscopic fenestration and biopsy are effective and confirm the diagnosis.

**Conclusion:**

This instance of a rare glioependymal cyst located in the midline, spanning the suprasellar and retrosellar regions, is an uncommon occurrence. Its sole presentation was precocious puberty. The successful management of this condition was achieved through an endoscopic approach, leading to the normalization of endocrine abnormalities.

## Highlights

• Glial cysts are extremely rare
• Usually confused with arachnoidal cyst and confirmed by biopsy
• Reported to happen laterally in the brain
• Unique case of midline causing only hormonal deficit
• Treatment by endoscopy was excellent

## 1 Introduction

Also known as neuroglial cysts, glioependymal cysts are benign neuroepithelial lesions that are exceptionally uncommon and have been sporadically documented. These developmental anomalies can occur anywhere within the brain and spinal cord but are exceedingly rare, accounting for <1 % of primary intracranial cysts [1,2]. Glioependymal cysts can manifest in various locations, including brain tissue (with a predilection for the frontal lobe), ventricles, the subarachnoid space, cranial nerves such as the third nerve (III), the cerebellum, and the spinal cord.

## 2 Clinic

This work has been reported in line with the SCARE criteria [3]. A 7-year-old girl presented with an unusual instance of early-onset puberty. Her parents had observed substantial breast development and increased hair growth in her armpit and genital areas within the last three months. Notably, she did not experience any accompanying symptoms such as headaches, nausea, vomiting, or vision issues. A review of her growth chart indicated that her weight and height had not exhibited significant acceleration over the past year, consistently remaining in the 90th percentile. Additionally, a genital examination indicated Tanner stage 3 development.

Importantly, an ophthalmological assessment did not reveal any abnormalities (with a notable absence of papilledema). Her endocrinological profile revealed an increase of luteinizing hormone levels (5.26 mIU/ml) and follicle-stimulating hormone levels (10.50 mIU/ml). Her levels of thyroid-stimulating hormone and free thyroxine 4 were slightly low, as shown in Table 1. Magnetic resonance imaging (MRI) revealed a cystic space-occupying lesion measuring 35 × 52 × 52 mm^3^ within the third ventricle. This lesion exhibited isointensity to cerebrospinal fluid (CSF), appearing hyperintense in T2-weighted images (T2WI) and hypointense in T1-weighted images (T1WI) and FLAIR images.

**Table 1:** Hormonal tests pre and post-surgery.

	Preoperatively	Postoperatively
TSH (0.66–5.00 μ IU/ml)	0.65	2.25
FT4 (0.8–1.90 Pg/dl)	0.67	1.1
FSH (prepubertal up to 5.0 miU/ml)	10.50	1.65
LH (prepubertal 0–4.0 mlU/ml)	5.26	2.96
ACTH (7.2–63.3 pg/ml)	8.77	8.00
Cortisol 8 am (5.5–28.7 μg/dl)	13.10	1.28

**Fig. 1:**
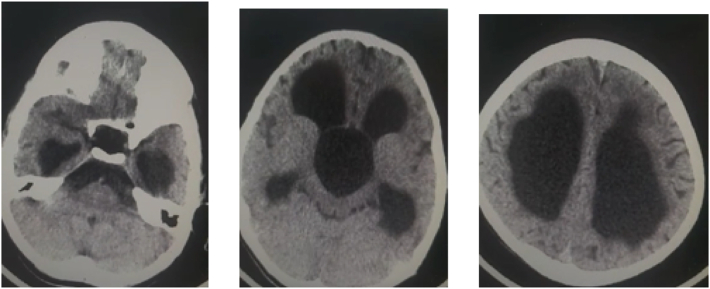
Non-contrast CT showing cystic lesion within 3rd ventricle causing severe hydrocephalus.

**Fig. 2:**
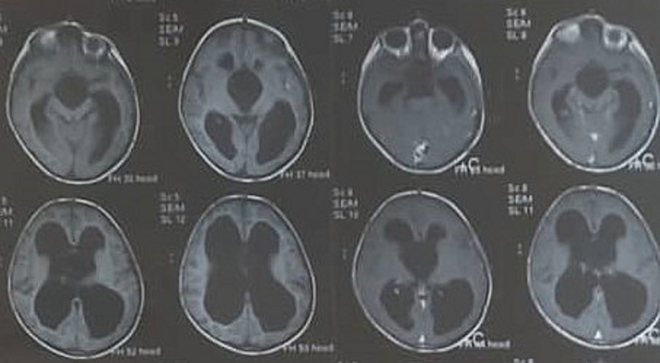
Pre- and post-contrast T1WI sagittal MRI showing large non-enhancing cyst within 3rd ventricle herniated into suprasellar and prepontine cisterns.

**Fig. 3:**
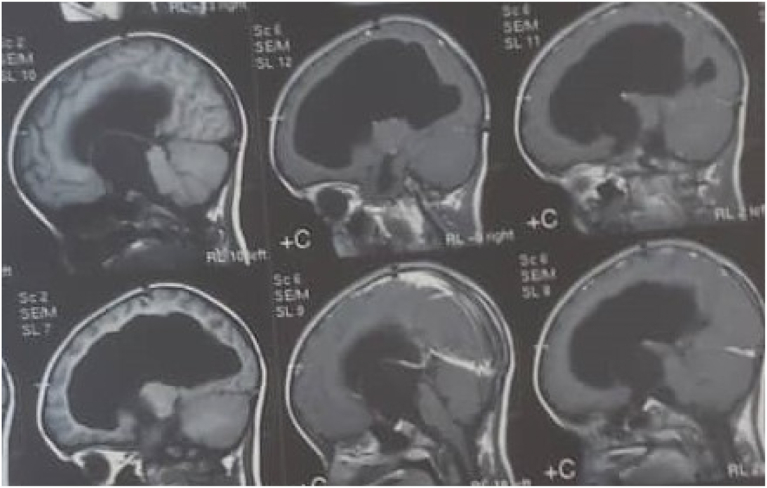
Axial T1WI pre- and post-contrast MRI showing non-enhancing cyst within 3rd ventricle.

Notably, both a computed tomography (CT) scan and MRI displayed a very thin and smooth wall surrounding the cyst, and there was no evidence of abnormal enhancement following contrast injection. This cyst caused dilatation in the lateral ventricles with no evident transependymal edema in the surrounding area. The aqueduct and fourth ventricle displayed no remarkable abnormalities. Additionally, the lesion extended along the clivus and protruded into the prepontine cistern (Fig. 1, Fig. 2, Fig. 3). Importantly, there was no apparent tortuosity of the optic nerves, which made it less likely that intracranial hypertension would be a contributing factor. The radiological investigation presented a dilemma in the differential diagnosis, which encompassed considerations such as an arachnoid cyst or a glioependymal cyst.

**Fig. 4:**
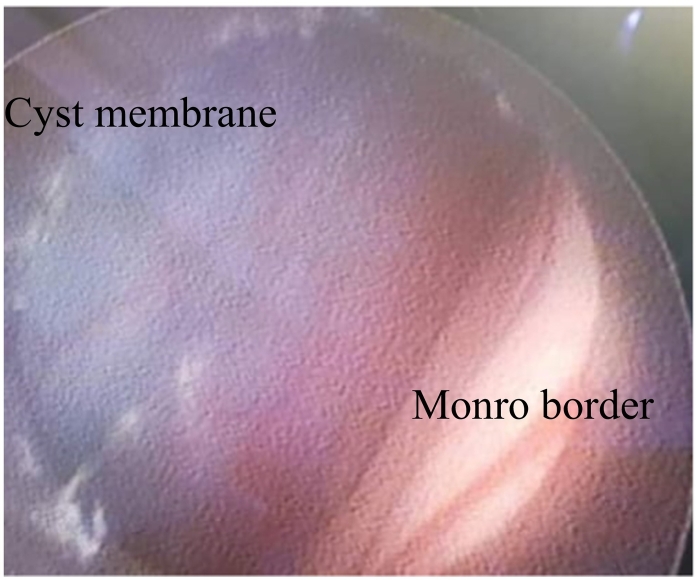
Endoscopic intra-ventricular view of the superior membrane of the glioependymal cyst through foramen of Monro.

**Fig. 5:**
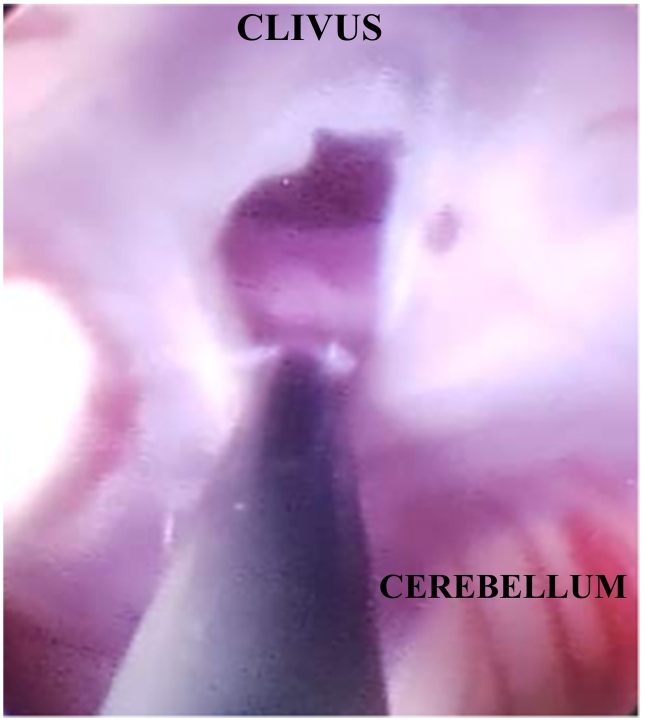
Fenestration at the deeper membrane of the cyst toward the pre-pontine cistern.

Brain endoscopy was conducted with general anesthesia. Surprisingly, the ventricular tape did not indicate elevated ventricular pressure on its own. The cyst was observed at the level of the foramen of Monro. We performed multiple biopsies in attempts to create a large hole in the cyst wall. As we penetrated the cyst, the liquid inside appeared very clear and similar to CSF. While navigating behind the sella dorsum, we successfully fenestrated the cyst and had a clear view of the basilar artery along with its bifurcation (Fig. 4, Fig. 5).

**Fig. 6:**
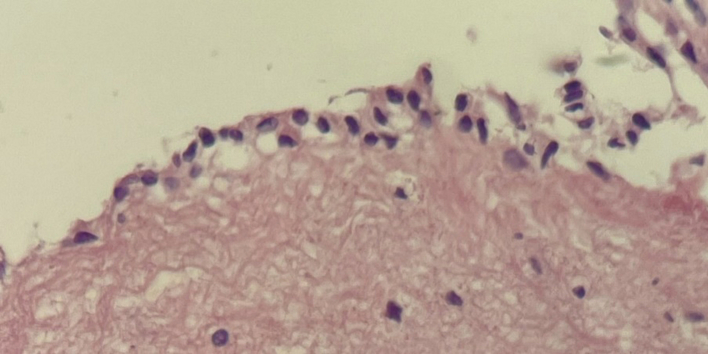
Photomicrograph showing the cyst wall lined by a single layer of columnar epithelium. The wall is composed of normal glial tissue (hematoxylin and eosin, 40×).

MRI was done postoperatively and showed only a slight decrease in the cyst volume. The endocrinological profile revealed an early normalization of the tested hormones in the morning, and cortisol levels significantly dropped (Table 1). The histopathological examination affirmed the diagnosis of a glioependymal cyst, which is supported by the findings illustrated in (Fig. 6). This examination involved a comprehensive analysis of the cyst's tissue composition, cellular characteristics, and structural features, all of which were consistent with the distinctive attributes typically associated with glioependymal cysts.

## 3 Discussion

Glioependymal cysts are a rare type of brain lesion that have been the subject of limited research. A review of PubMed revealed fewer than 30 reported cases of this pathology. Glioependymal cysts can manifest in various regions of the central nervous system, including the intraparenchymal region [2,[4], [5], [6], [7], [8], [9], [10], [11], [12], [13], [14], [15]], ventricles, subarachnoid space [[16], [17], [18], [19]], and occasionally within nerves [20] and the spinal cord [1,21,22]. There are also a few reports of cases in the posterior fossa and brain stem [[23], [24], [25], [26], [27], [28], [29], [30]]. These cysts originate from embryonic remnants and constitute <1 % of all cystic brain lesions [2].

The embryological genesis of these cysts is well defined. During the neural groove stage and immediately following tube closure, neuroepithelial cells undergo rapid division, resulting in the formation of the neuroepithelial layer [31]. Upon complete closure of the neural tube, neuroepithelial cells begin to differentiate into two distinct cell types. One type is characterized by a large round nucleus with a pale nucleoplasm and a dark-staining nucleolus and develops into primitive nerve cells or neuroblasts. The alternative pathway taken by neuroepithelial cells leads to their transformation into ependymal cells, which line the ventricular spaces and the central canal [32].

The prevailing theory concerning the origin of ependymal cysts suggests that the floor plate of the neural tube protrudes on the ventral side and becomes isolated, ultimately forming an ependymal cyst [19,33]. At the macroscopic level, these cysts are encased in a thin, translucent membrane, which is filled with a fluid that closely resembles CSF, although it may exhibit high protein content [33]. The cyst wall comprises an inner layer of ependymal cells with secretory activity, which accounts for the gradual growth of the cyst [1].

The clinical manifestations associated with neuroglial cysts depend on their size and location. Patients may experience symptoms such as seizures, motor deficits, chronic headaches, psychomotor delays, or even macrocephaly in infants [[34], [35], [36]]. Occasionally, these cysts are discovered incidentally [24]. They can occur in isolation or alongside other brain abnormalities, of which agenesis of the corpus callosum is the most common [[36], [37], [38], [39], [40]]. Endocrine manifestations are also exceedingly rare [41].

In imaging studies, neuroglial cysts typically appear as large periventricular or interhemispheric lesions with similar density and signal characteristics to those of CSF when observed using CT and MRI. In certain cases, the fluid content may display a higher signal in T2 sequences, indicating a fluid rich in proteins. Importantly, the cyst wall is not enhanced with the administration of gadolinium contrast [25]. Moreover, prenatal diagnosis can be achieved through morphological ultrasound, enabling close monitoring [24]. Surgical intervention is necessary for symptomatic patients, and various surgical approaches have been documented in the literature [23,42]. One commonly employed technique with minimal morbidity is neuroendoscopic cyst fenestration [26].

## 4 Conclusion

This case of a glioependymal cyst in the midline within the suprasellar and retrosellar region is extremely rare. Its only manifestation was precocious puberty. The management through an endoscopic approach was highly successful and resulted in the normalization of endocrine abnormalities. Glioependymal cysts should be differentiated from other types of cysts, such as arachnoid cysts. This differentiation can primarily occur through biopsy during endoscopy. The clinical results may be closely related to the specific histology of the cyst, but it needs to be verified.

## Consent

Written informed consent was obtained from the patient for publication of this case report and accompanying images. A copy of the written consent is available for review by the Editor-in-Chief of this journal on request.

All informed consents were obtained from all subjects and/or their legal guardian(s).All participants were aware of the study's purpose, risks, and benefits.

## Ethical approval

All approvals for this study were taken from “ethics committee” at children's university hospital and faculty of medicine at Damascus University. [3022.23] DEC22ed 2023.

## Funding

None.

## Author contribution

Hassan Kadri wrote the manuscript/data

Mazen Dughly wrote the radiology manuscript

Ghiath Hamed wrote the pathology manuscript

Raed Abouharb supervision

Rostom Mackieh revision

Sameer Bakleh revision

## Guarantor

Dr Hassan Kadri.

## Research registration number

N/A.

## Conflict of interest statement

None.
